# The crystal structure of titanium dioxide nanoparticles influences immune activity in vitro and in vivo

**DOI:** 10.1186/s12989-018-0245-5

**Published:** 2018-01-30

**Authors:** Rob J. Vandebriel, Jolanda P. Vermeulen, Laurens B. van Engelen, Britt de Jong, Lisa M. Verhagen, Liset J. de la Fonteyne-Blankestijn, Marieke E. Hoonakker, Wim H. de Jong

**Affiliations:** 1 Centre for Health Protection, National Institute for Public Health and the Environment (RIVM), PO Box 1, 3720 BA Bilthoven, The Netherlands; 2 Intravacc, PO Box 450, 3720 AL Bilthoven, The Netherlands

**Keywords:** Titanium dioxide, Anatase, Rutile, Dendritic cell, Maturation, Inhalation, LgE, LgG1, Ovalbumin, Adjuvant

## Abstract

**Background:**

The use of engineered nanoparticles (NP) is widespread and still increasing. There is a great need to assess their safety. Newly engineered NP enter the market in a large variety; therefore safety evaluation should preferably be in a high-throughput fashion. In vitro screening is suitable for this purpose. TiO _2_ NP exist in a large variety (crystal structure, coating and size), but information on their relative toxicities is scarce. TiO _2_ NP may be inhaled by workers in e.g. paint production and application. In mice, inhalation of TiO _2_ NP increases allergic reactions. Dendritic cells (DC) form an important part of the lung immune system, and are essential in adjuvant activity. The present study aimed to establish the effect of a variety of TiO _2_ NP on DC maturation in vitro. Two NP of different crystal structure but similar in size, uncoated and from the same supplier, were evaluated for their adjuvant activity in vivo.

**Methods:**

Immature DC were differentiated in vitro from human peripheral blood monocytes. Exposure effects of a series of fourteen TiO _2_ NP on cell viability, CD83 and CD86 expression, and IL-12p40 and TNF-α production were measured. BALB/c mice were intranasally sensitized with ovalbumin (OVA) alone, OVA plus anatase TiO _2_ NP, OVA plus rutile TiO _2_ NP, and OVA plus Carbon Black (CB; positive control). The mice were intranasally challenged with OVA. OVA-specific IgE and IgG1 in serum, cellular inflammation in bronchoalveolar lavage fluid (BALF) and IL-4 and IL-5 production in draining bronchial lymph nodes were evaluated.

**Results:**

All NP dispersions contained NP aggregates. The anatase NP and anatase/rutile mixture NP induced a higher CD83 and CD86 expression and a higher IL-12p40 production in vitro than the rutile NP (including coated rutile NP and a rutile NP of a 10-fold larger primary diameter). OVA-specific serum IgE and IgG1 were increased by anatase NP, rutile NP, and CB, in the order rutile<anatase<CB. The three particles similarly increased IL-4 and IL-5 production by bronchial LN cells and eosinophils and lymphocytes in the BALF. Neutrophils were induced by rutile NP and CB but not by anatase NP.

**Conclusions:**

Our data show that measuring CD83 and CD86 expression and IL-12p40 and TNF-α production in DC in vitro may provide an efficient way to screen NP for potential adjuvant activity; future studies should establish whether this also holds for other NP. Based on antigen-specific IgE and IgG1, anatase NP have higher adjuvant activity than rutile NP, confirming our in vitro data. Other parameters of the allergic response showed a similar response for the two NP crystal structures. From the viewpoint of safe(r) by design products, rutile NP may be preferred over anatase NP, especially when inhalation exposure can be expected during production or application of the product.

**Electronic supplementary material:**

The online version of this article (10.1186/s12989-018-0245-5) contains supplementary material, which is available to authorized users.

## Background

The use of engineered nanoparticles (NP) is widespread and still increasing. Therefore, there is great need to assess their effect on human health. Newly developed NP enter the market frequently and in a large variety, therefore safety evaluation should preferably provide results quickly and in a high-throughput fashion. An in vitro screening assay with demonstrated predictive value is suitable for this purpose. A further advantage of such an approach is that it reduces the use of animals, as animal experiments can be designed using knowledge obtained from in vitro experiments [1]. TiO_2_ NP are one of the most frequently used nanomaterials in paints; both production and application may result in inhalation exposure. Following inhalation TiO_2_ NP are known to be able to enhance an allergic response (adjuvant activity) [2–5] via the NF-κB pathway [6]. Dendritic cells (DC) form an important part of the lung immune system [7]. DC maturation is an essential step in the adaptive immune response [8] and plays an important role in enhancing an allergic response after inhalation of diesel soot particles [9, 10] and particulate matter [11] and likely also of TiO_2_ NP. In fact, TiO_2_ NP have been shown to induce DC maturation [12] via the NF-κB pathway [13]. Therefore, in the present study DC maturation is used as in vitro screening assay to determine the activation potency of TiO_2_ NP.

A series of TiO _2_ NP with different crystal structure, coating, and size were evaluated in the DC maturation assay in order to evaluate their DC activation potency as measure for their safety from the viewpoint of safe(r) by design products, especially when inhalation exposure can be expected during production or application of the product. Two NP of different crystal structure but similar in size, uncoated and from the same supplier, were evaluated for their adjuvant activity in vivo.

## Results

### Particle characterization

#### Size of the nanoparticles in dispersion

Table 1 lists the fourteen NP tested including their primary size and their size in dispersion (in 0.05% BSA in H_2_O). The mean size of the various NP was between 157 and 212 nm, suggesting that, with the exception of the rutile NP of 200 nm, in suspension all NP were aggregated. The median size (the highest point in the peak of the size distribution) of the various NP was between 84 and 175 nm, and showed more variation between the various NP than the mean size.

**Table 1:** List of TiO _2_ NP used in this study, including their size in dispersion

Manu-facturer	identification or product #	crystal form ^a^	primary size ^a^ (nm)	SSA ^a^ (m^2^/g)	purity ^a^ (%)	coating ^a^	mean ± SD (nm)	median ± SD (nm)
JRC	NM-102	anatase	20	90	N/A	none	182.6 ± 21.1	86.6 ± 30.8
JRC	NM-103	rutile	20	60	N/A	hydrophobic	157.4 ± 8.9	102.0 ± 49.2
JRC	NM-104	rutile	20	60	N/A	hydrophilic	162.4 ± 5.8	147.4 ± 14.5
Skyspring	7910DL	anatase	10–25	50–150	99.5	none	186.4 ± 14.2	175.4 ± 29.7
Skyspring	7918DL	anat./rut.	10–30	50–100	99.5	none	169.6 ± 7.9	129.4 ± 38.9
Skyspring	7920DL	rutile	10–30	≈50	99.5	none	169.6 ± 25.7	139.8 ± 47.3
Skyspring	7923DL	rutile	20–40	> 40	99	SiO_2_	177.2 ± 40.9	84.2 ± 17.8
Skyspring	7925DL	rutile	20–40	> 40	99	Al_2_O_3_	194.0 ± 15.9	128.8 ± 57.2
Io-Li-Tec	NO-0038-HP	anatase	20	> 120	99.5	none	208.6 ± 22.0	144.6 ± 42.0
Io-Li-Tec	NO-0046-HP	rutile	10–30	≈50	99.5	none	170.6 ± 13.2	139.2 ± 10.6
Io-Li-Tec	NO-0051-HP	rutile	200	8	99.5	none	212.4 ± 22.1	140.2 ± 61.5
Io-Li-Tec	NO-0058-HP	anatase	10–25	50–150	99.5	none	157.4 ± 28.3	121.2 ± 35.5
Io-Li-Tec	NO-0065-HP	rutile	20–40	40	99	silicon oil	161.6 ± 9.5	153.0 ± 2.8
Io-Li-Tec	NO-0066-HP	rutile	20–40	> 40	99	SiO_2_	175.8 ± 19.0	157.0 ± 5.7

*SSA* specific surface area, *N/A* not available

^a^Information form the manufacturer or, in case of JRC [34], the supplier

#### Endotoxin content of the nanoparticles

Using the endoLISA kit an endotoxin concentration in the dispersion solution without NP of 52 EU/ml was found, while the endotoxin concentration in the dispersions containing NP ranged between 0 and 84 EU/ml. Multi-group ANOVA indicated no relationship between crystal structure of the NP and their endotoxin concentration (*P* = 0.46).

### In vitro study

#### Exposure effect on viability

WST-1 staining indicated no loss of viability for the highest NP concentration tested (128 μg/ml; Additional file 1: Table S1). Live dead staining was performed to gate viable cells and to evaluate possible NP exposure effects on viability. No clear dose-related effects were seen for any of the NP. For some NP, two or more consecutive doses induced staining levels below 80% of the non-exposed controls (10–30 nm uncoated anatase/rutile mixture, 20–40 nm SiO_2_ coated rutile, 20–40 nm Al_2_O_3_ coated rutile, and 20–40 nm silicon oil coated rutile NP); these staining levels never fell below 50%. Additional file 1: Table S2 shows the results of live dead staining for the highest NP concentration tested.

#### Exposure effect on surface marker expression

Maturation of DC leads to an increased expression of surface markers, such as CD40, CD80, CD83, CD86 and HLA-DR [8]. In order to determine whether exposure to the different NP leads to DC maturation, the effect on the expression of these markers was measured using flow cytometry. Next to the aforementioned markers, as a control the expression was measured of CD14, a surface marker found on monocytes but not on DC. During culture of DC from monocytes, CD14 expression should disappear. After maturation, CD14 expression was found to be low and not affected by NP exposure. This protocol has consistently shown a clear upregulation by LPS of CD40, CD80, CD83, CD86 and HLA-DR [14].

For all NP tested exposure resulted in a dose-dependent effect on the mean fluorescence index (MFI) of CD83 and CD86, while no exposure effects on the MFI of CD40, CD80 and HLA-DR were seen.

In order to make a comparison between the NP in their capacity to induce DC maturation the MFI of CD83 at the highest NP concentration tested was divided by that of the blank control and the NP were ranked according to these ratios (Additional file 1: Table S3). The results show that anatase and anatase/rutile NP have a higher CD83 inducing capacity than rutile ones. Multi-group ANOVA indicated a statistically significant difference between the crystal structures (*P* = 0.00013).

For CD86 a similar approach was taken as for CD83 (Additional file 1: Table S4). Anatase and anatase/rutile NP have a higher CD86 inducing capacity than rutile ones, except for Skyspring SiO_2_ coated rutile NP. Multi-group ANOVA indicated a statistically significant difference between the crystal structures (*P* = 0.00697). The range in CD83 induction was almost twice that of CD86 (4.5 versus 2.4).

#### Exposure effect on cytokine production

The induction of IL-6, IL-8, IL-10, IL-12p40, IL-12p70 and TNF-α production was measured. The production of IL-10 and IL-12p70 was lower than the detection limit, while IL-8 production did not show a dose-response relationship. IL-6, IL-12p40 and TNF-α showed a dose-response relationship and the NP were ranked, using the same approach as described above: production at the highest NP concentration tested was divided by that of the blank control, and the NP were ranked according to these ratios.

Ranking the NP according to the ratio of IL-12p40 induction shows that the anatase NP (including the anatase/rutile NP) more strongly induced IL-12p40 production than the rutile NP (Additional file 1: Table S5). Multi Group ANOVA indicated a statistically significant difference between the crystal structures (*P* = 0.01256).

Ranking the NP according to the ratio of TNF-α induction shows that the anatase NP (including the anatase/rutile NP) more strongly induced TNF-α production than the rutile NP, except for Io-Li-Tec uncoated 200 nm NP (Additional file 1: Table S6). Multi Group ANOVA indicated that this difference between the crystal structures was, however, not statistically significant (*P* = 0.24924).

IL-6 induction showed no consistent relation with crystal structure, coating or manufacturer (Additional file 1: Table S7). Multi-group ANOVA indicated no statistically significant difference between the crystal structures (*P* = 0.99891).

Table 2 shows the ranking based on CD83, CD86, and IL-12p40. Using Support Vector Machines on the combined results on CD83, CD86, and IL-12p40, the accuracy of classification of the crystal structure was found to be 100%. Classification of coating or manufacturer did not result in a prediction that was better than a random prediction.

**Table 2:** Ranking of TiO _2_ NPs based on induction of CD83 and CD86 expression, and IL-12p40 production. 1, strongest induction; 14, weakest induction. The ranks for the three parameters were summed and the TiO _2_ NPs were ranked accordingly

crystal form	primary size (nm)	coating	manufacturer	CD83	CD86	IL-12p40	score
anat./rut.	10–30	none	Skyspring	1	1	3	5
anatase	20	none	JRC	3	2	1	6
anatase	10–25	none	Io-Li-Tec	4	5	2	11
anatase	20	none	Io-Li-Tec	5	4	4	13
anatase	10–25	none	Skyspring	2	**6**	5	13
rutile	20–40	SiO_2_	Skyspring	6	**3**	9	18
rutile	20–40	Al_2_O_3_	Skyspring	8	7	6	21
rutile	10–30	none	Skyspring	7	8	8	23
rutile	200	none	Io-Li-Tec	9	9	7	25
rutile	20	hydrophobic	JRC	12	11	10	33
rutile	10–30	none	Io-Li-Tec	14	10	11	35
rutile	20–40	SiO_2_	Io-Li-Tec	10	13	13	36
rutile	20	hydrophilic	JRC	11	12	14	37
rutile	20–40	silicon oil	Io-Li-Tec	13	14	12	39

Bold: ranking does not fit the anatase vs. rutile difference in induction

### In vivo study

#### Serum immunoglobulins

##### IgE

In the treatment groups where OVA was administered only during the challenge phase, OVA-specific IgE was 14 ng/ml; NP exposure during the “sensitization” phase did not affect this level (results not shown).

**Fig. 1:**
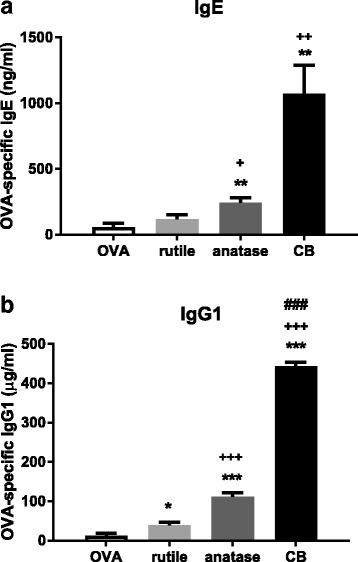
**a**. OVA-specific IgE in serum. **b**. OVA-specific IgG1 in serum. Mice were sensitized with OVA alone, OVA + rutile TiO_2_ NP, OVA + anatase TiO_2_ NP, or OVA + Carbon Black (CB), and challenged with OVA. *N* = 6, mean ± SEM is shown. (*), (**), and (***) *P* < 0.05, *P* < 0.01, and *P* < 0.001vs. OVA alone; (+), (++), and (+++) *P* < 0.05, *P* < 0.01, and *P* < 0.001 vs. OVA + rutile NP; (###) *P* < 0.001 vs. OVA + anatase NP

When OVA was administered during both the sensitization and challenge phase, OVA-specific IgE was 4 times higher relative to the animals that received OVA only during the challenge phase. Co-exposure to OVA and anatase TiO_2_ NP, or OVA and the positive control Carbon Black (CB) during the sensitization phase increased OVA-specific IgE (*P* < 0.01; Fig. 1a). For rutile TiO_2_ NP the increase was small and not statistically significant; OVA-specific IgE was 2 times higher after anatase NP co-exposure compared to rutile NP co-exposure (*P* < 0.05). These results may suggest that anatase NP have adjuvant activity, whereas rutile NP do not.

##### IgG1

In the treatment groups where OVA was administered only during the challenge phase, OVA-specific IgG1 was 74 ng/ml; NP exposure during the “sensitization” phase did not affect this level (results not shown).

When OVA was administered during both the sensitization and challenge phase, OVA-specific IgG1 was 160 times higher relative to the animals that received OVA only during the challenge phase. Co-exposure to OVA and rutile TiO_2_ NP, OVA and anatase TiO_2_ NP, or OVA and CB increased OVA-specific IgG1 (*P* < 0.05 for rutile NP; *P* < 0.001 for anatase NP and CB; Fig. 1b). OVA-specific IgG1 was 3 times higher after anatase NP co-exposure compared to rutile NP co-exposure (*P* < 0.001). These results may suggest that both rutile and anatase TiO_2_ NP have an adjuvant activity and that the adjuvant activity is greater for anatase NP than for rutile NP.

#### Cytokines produced by bronchial lymph node cells and spleen cells

In the supernatants of the bronchial lymph node (LN) and spleen cell cultures IFN-γ, IL-1β, IL-4, IL-5, IL-17A, MCP-1 and TNF-α were measured.

##### Bronchial lymph node cells

In the treatment groups where OVA was administered only during the challenge phase, production of IL-1β, IL-17 and MCP-1 was found not to be consistently above the detection limit. For the other cytokines, no treatment–related effects were seen.

**Fig. 2:**
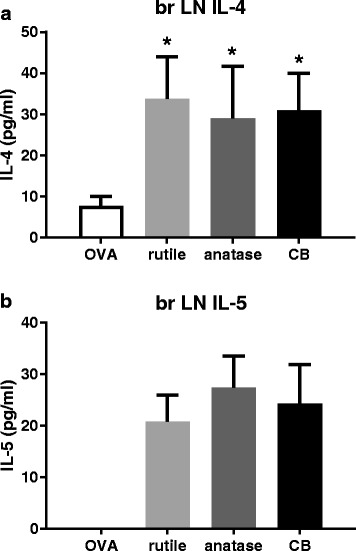
**a**. IL-4 production by LN cells. **b**. IL-5 production by LN cells. Mice were sensitized with OVA alone, OVA + rutile NP, OVA + anatase NP, or OVA + CB, and challenged with OVA. LN cell preparations were made and incubated with Con A for 24 h. N = 6, mean ± SEM is shown. (*) *P* < 0.05 vs. OVA alone

In the treatments groups where OVA was administered during both the sensitization and challenge phase, production of IL-1β and MCP-1 was found not to be consistently above the detection limit. For IFN-γ, IL-17A and TNF-α, no treatment-related effects were seen. In the supernatants of the OVA-alone animals the IL-4 concentration was 8 pg/ml. Co-exposure to OVA and rutile TiO_2_ NP, OVA and anatase TiO_2_ NP, or OVA and CB resulted in a 4-fold increase in IL-4 levels (*P* < 0.05) compared to OVA-alone (Fig. 2a). IL-5 was absent from the supernatants of the OVA-alone animals. In the supernatants of the animals co-exposed to OVA and rutile NP, OVA and anatase NP, or OVA and CB, the IL-5 concentration was 24 pg/ml (Fig. 2b).

##### Spleen cells

For spleen cells no effect of NP treatment was observed.

#### Cells in the lung lavage

##### Eosinophils

**Fig. 3:**
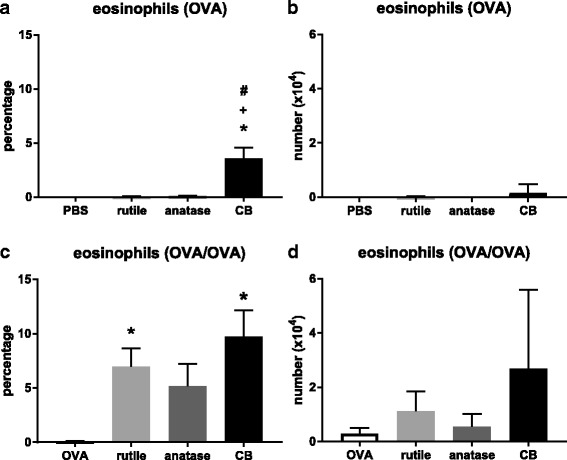
**a**. Percentage of eosinophils in the BALF after OVA challenge. **b**. Number of eosinophils in the BALF after OVA challenge. **c**. Percentage of eosinophils in the BALF after OVA sensitization and challenge. **d**. Number of eosinophils in the BALF after OVA sensitization and challenge. A, B. Mice were sensitized with PBS, rutile NP, anatase NP, or CB, and challenged with OVA. C, D. Mice were sensitized with OVA alone, OVA + rutile NP, OVA + anatase NP, or OVA + CB, and challenged with OVA. N = 6, mean ± SEM is shown. In A and B, (*) *P* < 0.05 vs. PBS alone; (+) *P* < 0.05 vs. rutile NP; (#) *P* < 0.05 vs. anatase NP. In C and D, (*) *P* < 0.05 vs. OVA alone

An infiltrate of eosinophils is suggestive of an allergic inflammatory response. For the treatment groups where OVA was administered only during challenge, the lungs of the control animals did not show eosinophils, whereas the animals that were exposed to rutile NP and anatase NP showed a low percentage of eosinophils (Fig. 3a). CB exposed animals showed a higher percentage of eosinophils compared to rutile NP and anatase NP exposed animals (*P* < 0.05). When the effects were expressed as number of eosinophils a similar pattern was found but without statistical significance (Fig. 3b).

The treatment groups that were both sensitized and challenged to OVA showed a considerably higher percentage of eosinophils compared to the groups where OVA was administered only during challenge (Fig. 3c). For these groups the percentage of eosinophils was higher after co-exposure to OVA and rutile NP, and OVA and CB, compared to OVA alone (*P* < 0.05). When the effects were expressed as number of eosinophils a similar pattern was found but without statistical significance (Fig. 3d).

##### Lymphocytes

**Fig. 4:**
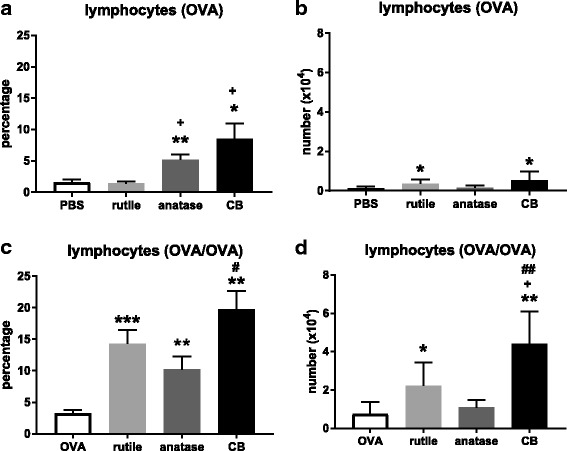
**a**. Percentage of lymphocytes in the BALF after OVA challenge. **b**. Number of lymphocytes in the BALF after OVA challenge. **c.** Percentage of lymphocytes in the balf after ova sensitization and challenge. **d**. Number of lymphocytes in the BALF after OVA sensitization and challenge. See legend to Fig. 3. In A and B, (*) and (**) *P* < 0.05 and *P* < 0.01 vs. PBS alone; (+) *P* < 0.05 vs. rutile NP. In C and D, (*), (**) and (**) *P* < 0.05, *P* < 0.01 and *P* < 0.01 vs. OVA alone; (+) *P* < 0.05 vs. OVA + rutile NP; (##) *P* < 0.05 vs. OVA + anatase NP

An infiltrate of lymphocytes is suggestive of a chronic inflammatory response. For the treatment groups where OVA was administered only during challenge, exposure to anatase NP and CB increased the percentage of lymphocytes in the lungs (*P* < 0.01 and *P* < 0.05, respectively; Fig. 4a). Exposure to anatase NP and CB resulted in a higher percentage of lymphocytes compared to rutile NP (*P* < 0.05). Rutile NP and CB exposure resulted in an increased number of lymphocytes (Fig. 4b).

The treatment groups that were both sensitized and challenged to OVA showed a higher percentage of lymphocytes compared to the groups where OVA was administered only during challenge (Fig. 4c). For these groups the percentage of lymphocytes was higher after co-exposure to OVA and rutile NP, OVA and anatase NP, and OVA and CB, compared to OVA alone (*P* < 0.001, *P* < 0.01, and *P* < 0.01, respectively). Co-exposure to OVA and CB resulted in a higher percentage of lymphocytes compared to co-exposure to OVA and anatase NP (*P* < 0.05). Co-exposure to OVA and rutile NP, and OVA and CB resulted in an increased number of lymphocytes (*P* < 0.05 and *P* < 0.01, respectively; Fig. 4d). Co-exposure to OVA and CB resulted in an increased number of lymphocytes compared to co-exposure to OVA and rutile NP, and OVA and anatase NP (*P* < 0.05 and *P* < 0.01, respectively).

##### Neutrophils

An infiltrate of neutrophils is suggestive of a non-allergic, acute inflammatory response.

**Fig. 5:**
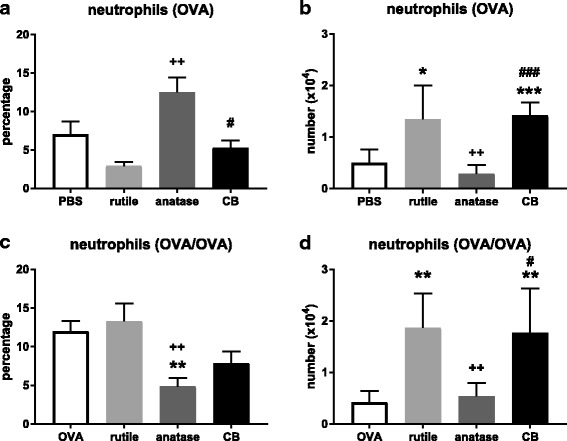
**a**. Percentage of neutrophils in the BALF after OVA challenge. **b**. Number of neutrophils in the BALF after OVA challenge. **c**. Percentage of neutrophils in the BALF after OVA sensitization and challenge. **d**. Number of neutrophils in the BALF after OVA sensitization and challenge. See legend to Fig. 3. In A and B, (*) and (***) *P* < 0.05 and *P* < 0.001 vs. PBS alone; (++) *P* < 0.01 vs. rutile NP; (#) and (###) *P* < 0.05 and *P* < 0.001 vs. anatase NP. In C and D, (**) *P* < 0.01 vs. OVA alone; (++) *P* < 0.01 vs. OVA + rutile NP; (#) *P* < 0.05 vs. OVA + anatase NP

For the treatment groups where OVA was administered only during challenge, exposure to anatase NP resulted in a higher percentage of neutrophils in the lungs (*P* < 0.01; Fig. 5a). Exposure to CB resulted in a lower percentage of neutrophils compared to anatase NP (*P* < 0.05). Exposure to rutile NP and CB resulted in an increased number of neutrophils (*P* < 0.05 and *P* < 0.001, respectively; Fig. 5b). Exposure to anatase NP resulted in a smaller number of neutrophils compared to rutile NP and CB (*P* < 0.01 and *P* < 0.05, respectively).

The treatment groups that were both sensitized and challenged to OVA showed a rather similar percentage of neutrophils compared to the groups where OVA was administered only during challenge (Fig. 5c). Co-exposure to OVA and anatase NP resulted in a lower percentage of neutrophils compared to OVA alone, and to co-exposure to OVA and rutile NP (*P* < 0.01). Co-exposure to OVA and rutile NP and to OVA and CB resulted in a higher number of neutrophils compared to OVA alone (*P* < 0.01; Fig. 5d). Co-exposure to OVA and anatase NP resulted in a smaller number of neutrophils compared to OVA and rutile NP, and OVA and CB (*P* < 0.01 and *P* < 0.05, respectively).

##### Macrophages

**Fig. 6:**
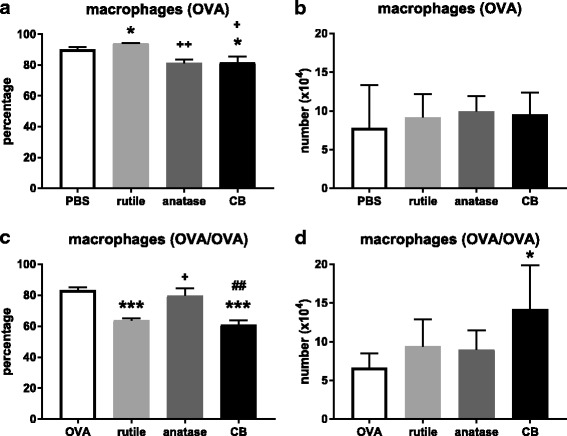
**a**. Percentage of macrophages in the BALF after OVA challenge. **b**. Number of macrophages in the BALF after OVA challenge. **c**. Percentage of macrophages in the BALF after OVA sensitization and challenge. **d**. Number of macrophages in the BALF after OVA sensitization and challenge. See legend to Fig. 3. In A and B, (*) *P* < 0.05 vs. PBS alone; (+) and (++) *P* < 0.05 and *P* < 0.01 vs. rutile NP. In C and D, (***) *P* < 0.001 vs. OVA alone; (+) *P* < 0.05 vs. OVA + rutile NP; (##) *P* < 0.01 vs. OVA + anatase NP

Macrophages are the dominant cell type in the lung lavage and their numbers are in general relatively constant; a decrease in their percentage is often due to an increase in the percentage of neutrophils, lymphocytes and eosinophils. For the treatment groups where OVA was administered only during challenge, exposure to rutile NP resulted in a higher percentage of macrophages in the lungs (*P* < 0.05; Fig. 6a), whereas exposure to CB resulted in a lower percentage (*P* < 0.05). Exposure to anatase NP and CB resulted in a lower percentage of macrophages compared to rutile NP (*P* < 0.01 and *P* < 0.05, respectively). No treatment related effects on the number macrophages was seen (Fig. 6b).

The treatment groups that were both sensitized and challenged to OVA showed a somewhat lower percentage of macrophages compared to the groups where OVA was administered only during challenge (Fig. 6c). The percentage of macrophages was lower after co-exposure to OVA and rutile NP, and to OVA and CB, compared to OVA alone (*P* < 0.001). The percentage of macrophages was higher after co-exposure to OVA and anatase NP compared to co-exposure to OVA and rutile NP and to OVA and CB (*P* < 0.05 and *P* < 0.01, respectively). This is likely due to a higher percentage of neutrophils (in case of rutile NP) and lymphocytes (in case of rutile NP and CB). Due to the small percentage of eosinophils in general, effects on these percentages have a minor influence on the percentage of macrophages. The number of macrophages was increased after co-exposure to OVA and CB compared to OVA alone (*P* < 0.05; Fig. 6d).

Overall, for eosinophils and lymphocytes no clear difference in the response to rutile NP and anatase NP is seen. For neutrophils, the response to rutile NP is higher than to anatase NP.

## Discussion

Here we have shown that in vitro anatase and anatase/rutile TiO _2_ NP induced a higher expression of CD83 and CD86 and a higher production of IL-12p40, than rutile NP, suggesting that DC maturation is induced to a greater extent by anatase and anatase/rutile NP than by rutile NP.

No effect of the size of the primary NP, their coating, and their manufacturer was found. The primary size of the NP is 10–40 nm, with the exception of one product having a primary particle size of 200 nm. NanoSight measurements showed that during cell culture all NP had a rather similar size distribution with a mean size of 160–210 nm. This suggests that, with the exception of the 200 nm particle, all NP showed aggregation and/or agglomeration. This may be an explanation for the absence of a size effect on the test results. It cannot be ruled out, however, that some of the coatings play a possible role in DC maturation. For instance, SiO_2_ NP have been shown to induce DC maturation [15, 16].

Different responses to anatase and rutile TiO_2_ NP have been reported earlier. Anatase NP induced a higher IL-8 production than rutile NP in A549 human lung epithelial cells [17]. Anatase and anatase/rutile NP induced a higher amount of reactive oxygen species (ROS) than rutile NP in a cell-free system [18]. Anatase NP induced stronger glutathione depletion and a greater reduction of superoxide dismutase than rutile NP in PC12 neuronal cells [19]. In this study, only anatase NP showed an increase in malondialdehyde; this molecule is formed by ROS from unsaturated fatty acids. Intranasal instillation of mice with 155 nm anatase NP resulted in higher IL-1β and TNF-α levels in the brain compared to similar treatment with 80 nm rutile NP [20]. In contrast to the previous studies, rutile NP induced higher ROS production than anatase NP in HEL30 keratinocytes [21]. These authors also found that rutile NP initiated cell death by apoptosis through formation of ROS, while anatase NP induced cell death by necrosis. In a co-culture of human blood vessel endothelial cells and DC, anatase NP induced a higher IL-1β, IL-10 and IFN-γ production than rutile NP [22]. Unlike our study, a similar expression of CD83 and CD86 on DC was found after exposure to anatase and rutile NP. In addition, they observed a similar induction of allogeneic naive CD4^+^ T-cells by DC that had been exposed to anatase and rutile NP. In conclusion, except for the studies by Braydich-Stolle et al. [21] and Schanen et al. [22], these studies are in line with our study suggesting that anatase and anatase/rutile TiO_2_ NP have a stronger adjuvant activity than rutile ones.

When comparing the responses between rutile and anatase NP for the various parameters, two markers of the allergic response, IgE and IgG1, are induced more strongly by anatase compared to rutile NP, whereas other markers for this response, IL-4, IL-5, eosinophils and lymphocytes are similarly induced by both NP, and neutrophils are induced more strongly by rutile compared to anatase NP. The conclusion of a stronger adjuvant activity of anatase NP compared to rutile NP should thus be made with some prudence.

The in vitro assay used here is generally accepted to measure effects on DC maturation. Since DC maturation is important in the induction of an adaptive immune response, and DC play an important role in the stimulation by particles of the adaptive immune response in the respiratory tract [10], the assumption was that stimulation of DC maturation in vitro might be translated to identify adjuvant activity for the immune response in vivo by NP, such as in the mouse ovalbumin allergy model. When inhaled, TiO_2_ NP can induce or enhance an allergic response (adjuvant activity) when co-administered with the allergen [2–5]. In our study two NP with different crystal structures but otherwise very similar, were selected to be tested in the mouse ovalbumin allergy model. The NP are of similar size (10–30 nm rutile NP, 10–25 nm anatase NP), are both uncoated, and are both from the same producer (Io-Li-Tec). By using the in vitro model prior to the experimental animal study, the number of NP for which testing in vivo was deemed relevant was limited to two.

In the animal study reported here we have shown that co-administration of an allergen (ovalbumin) and TiO_2_ NP, results, especially for the anatase TiO_2_ NPs in a marked adjuvant activity; these results are consistent with the in vitro findings using the DC model. Intratracheal instillation of rats of 80% anatase/20% rutile TiO_2_ NP but not of two rutile NP induced an increase in neutrophils and cytotoxicity in the bronchoalveolar lavage and proliferation of tracheobronchial epithelial cells and lung parenchymal cells [23]. Similar to our study, this study showed stronger effects of anatase NP compared to rutile NP on the rodent lungs. It should be noted, however, that this study did not involve an allergy model.

A more general question is whether physico-chemical properties of NP can provide information on the degree of oxidative stress, and thus glutathione depletion, DC maturation and allergic reactions. The higher ROS activity induced by anatase NP compared to rutile NP can be explained by their differences in surface chemistry [18]. Anatase is more suitable to adsorb oxygen in the form of O2^−^ and O^−^ than rutile TiO_2_ [24]. Water is bound by anatase as H^+^ and OH^−^, and by rutile as H_2_O [25, 26]. Both processes (adsorption of O2^−^ and O^−^; binding of H^+^ and OH^−^) facilitate ROS formation [24]. Glutathione (GSH) is an essential antioxidant that protects against oxidative stress. GSH in the cell decreases from exposure to oxidants [27]. GSH levels in antigen-presenting cells (such as DC) influence the Th1 versus the Th2 response; reduction in GSH levels leads to a decreased Th1 response [28]. The following chain of events may thus be suggested to explain differences for anatase vs. rutile TiO_2_ NP: higher adsorption of O2^−^ and O^−^, and stronger binding of H^+^ and OH^−^ on the surface of anatase NP → ROS ⇑ → glutathione depletion ⇑ → Th1 ⇓ → allergic Th2 response ⇑.

Li et al. [29] have shown that in an ovalbumin allergy model the response is determined by the oxidant potential of co-administered particulate matter. Our findings of a larger response due to co-administration of anatase NP compared to rutile NP are in line with this observation.

Relationships between physico-chemical properties of NP and biological effects have been established for band gap energy levels and cytotoxicity [30] and surface charge and lung fibrosis [31]. In this paper we established a relationship between crystal structure and induction of DC maturation as well as adjuvant activity.

The lower adjuvant activity of rutile TiO _2_ NP relative to anatase NP may be a reason to preferably apply rutile NP in order to reduce adjuvant activity during possible respiratory exposure. For a final choice of NP to be used additional NP characteristics should also be considered.

Finally, the in vitro DC maturation assay appears to be predictive for the adjuvant activity in vivo and may therefore be used as in vitro screening assay. However, this requires that first additional NP (multiple anatase and rutile NP, and also NP of other chemical identity such as SiO _2_ ) be compared in vitro and in vivo.

## Conclusions

In summary, we have shown that anatase TiO _2_ NP more strongly induce DC maturation than rutile NP; moreover, anatase NP show a stronger adjuvant activity in an in vivo allergy model. From the viewpoint of safe(r) by design products, rutile NP may be preferred over anatase NP, especially when inhalation exposure can be expected during production or application of the product The DC maturation assay used is a promising in vitro screen for adjuvant activity of NP.

## Methods

### In vitro studies

#### Materials

Fourteen TiO _2_ nanoparticles (NP) obtained from various suppliers (Joint Research Centre, Institute for Health and Consumer Protection, European Union; Skyspring Nano Materials Inc., Houston, TX, USA; Ionic Liquids Technologies GmbH, Heilbronn, Germany) were included in the study. They are listed in Table 1 .

#### NP dispersion and size determination

The NP (powder) were pre-wetted by adding a drop of absolute ethanol. The NP were then taken up in dispersion liquid (H_2_O + 0.05% BSA) to a concentration of 2.56 mg/mL. These suspensions were sonicated using a 450 W Digital Sonifier (Branson, Danbury, CT, USA) with 10% of the maximum energy for 16 min according to the Nanogenotox protocol [32]. The particle size was determined using Nanoparticle Tracking Analysis (Nanosight, Amesbury, UK), which is based on the Brownian movement of the NP. Each suspension was measured five times, filtered through a 0.45-μm filter and measured again five times. The particle size in the filtered suspensions is shown. It is expressed as median (size, to match the peak in the size distribution), and as an average, both ± SD of the five measurements.

#### Generation, exposure, and maturation of DC

Human-derived buffy coats were obtained from Sanquin (Amsterdam, the Netherlands). Peripheral blood mononuclear cells were isolated from buffy coats by density centrifugation (Lymphoprep; Axis Shield, Oslo, Norway). The cells were washed, harvested, and resuspended in RPMI-1640 (Gibco, Grand Island, NY, USA) supplemented with 2% heat-inactivated human serum (Harlan, Boxmeer, the Netherlands), 100 μg/mL streptomycin, 100 IU/mL penicillin, and 0.3 mg/mL L-glutamine. They were seeded in culture flasks (Corning, Amsterdam, the Netherlands) and were let to attach for 1 h. The cells were rinsed with warm (37°C) PBS and medium was added (RPMI-1640 supplemented with 10% heat-inactivated Foetal Calf Serum (“FCS”; Hyclone; GE Healthcare, Logan, UT, USA), streptomycin, penicillin, L-glutamine, 500 U/mL GM-CSF and 250 U/mL IL-4. At day 3, fresh cytokines were added. At day 6, the immature DC were harvested. Cell culture conditions were 37 °C in a humidified atmosphere containing 5% CO _2_ .

The DC were exposed to a concentration range of the TiO _2_ NP (0–128 μg/mL) for 48 h. After this, the viability of the cells was measured using the WST-1 assay. Next to staining for viability (“live-dead” staining), the expression of CD14, CD40, CD80, CD83, CD86, and HLA-DR was measured. To this end, the cells were washed twice with PBS and twice with FACS buffer (PBS pH 7.2, 0.5% BSA, 0.5 mM EDTA). To 100 μL of these cells, 100 μL staining mix 1 or staining mix 2 was added (see below). After incubation at 4 °C for 30 min, the cells were spun down, included in FACS buffer, and measured using the FACS Canto (Becton Dickinson Biosciences, Breda, the Netherlands)

**Table:** 

Marker	Label	Dilution
Staining mix 1		
CD80	FITC	1:40
CD14	PE	1:25
HLA-DR	Pacific Blue	1:1600
Live-dead	Aqua	1:200
Staining mix 2		
CD83	FITC	1:40
CD40	PE	1:20
CD86	Pacific Blue	1:800
Live-dead	Aqua	1:200

IL-12p40 was measured by ELISA (Becton Dickinson Biosciences) according to the manufacturer’s instructions. The other cytokines (IL-6, IL-8, IL-10, IL-12p70 and TNF-α) were measured using a Bio-Plex System (Bio-Rad, Veenendaal, the Netherlands).

#### Statistics

Each NP was tested at least three times; in Additional file 1: Tables S1-S7 a representative result is shown. To establish the significance of the difference in induction of CD83, CD86, IL-12p40, TNF-α and IL-6 between the groups of particles, a multi-group ANOVA was used for crystal structure, coating, and manufacturer. The analyses were run on a single surface marker or cytokine, versus the three categorical factors of crystal structure, coating and manufacturer, in one analysis. In addition, the accuracy of classification to the crystal structure was established using Support Vector Machines (SVM). Using the radial kernel on scaled data, SVM [33] creates a separating hyperplane. The rank data is the input. The crystal structure is binary, with anatase/rutile taken as anatase.

### In vivo study

#### Animals

Specific pathogen-free (SPF) female BALB/cAnNCrl mice [2], 6–8 weeks of age, were obtained from Charles River (Sulzfeld, Germany) and randomly assigned to a treatment group. Animals were bred under SPF conditions and barrier maintained during the experiment. Drinking water and conventional feed were provided ad libitum. Husbandry conditions were maintained according to all applicable provisions of the national laws, Experiments on Animals Decree and Experiments on Animals Act. The experiment was approved by an independent ethical committee (the Animal Experiments Committee of the National Institute for Public Health and the Environment) prior to the study.

#### Animal treatment and euthanasia

Uncoated 10–30 nm rutile TiO _2_ NP (“ILT46”; NO-0046-HP, Io-Li-Tec, Germany), uncoated 10–25 nm anatase TiO _2_ NP (“ILT58”; NO-0058-HP, Io-Li-Tec, Germany), and Carbon Black (“CB”, Printex 90, Degussa, Germany) were tested. They were dissolved in PBS to 6.67 mg/mL. Ovalbumin (“OVA”; Grade VII; Sigma-Aldrich, Zwijndrecht, the Netherlands) was dissolved in PBS to 10 mg/mL. Endotoxin was removed from OVA using a Detoxi-Gel Endotoxin Removing Column (Pierce; Thermo Fisher Scientific, Etten Leur, the Netherlands) according to the manufacturer’s instructions. For the OVA-alone group, 1 mL OVA was diluted with 1 mL PBS. To 1 mL of each of the NP suspensions, 1 mL PBS or 1 mL OVA was added. To each of the eight samples (PBS, OVA, ILT46, ILT58, CB, ILT46/OVA, ILT58/OVA, and CB/OVA), 100 μL mouse albumin (Sigma-Aldrich) was added. The samples were sonicated using a Digital Sonifier (Branson) with 10% of the maximum energy for 16 min. Animals were sensitized intranasally under deep isoflurane anaesthesia by adding 20 μL of the sample in each of the two nostrils (40 μL per animal; 0.45% OVA and 120 μg NP) at days 0, 1, and 2. Animals were challenged intranasally under deep isoflurane anaesthesia by adding 20 μL OVA in each of the two nostrils (40 μl per animal; 0.45% OVA) at days 25, 26, and 27.

At day 28, the animals were weighed. They were sacrificed by isoflurane euthanasia. Blood was collected (Greiner MiniCollect tubes) and the serum samples were stored at − 80 °C. The lungs were lavaged with PBS (1 mL per 25 g animal weight). This was repeated twice. Bronchoalveolar lavage (BAL) fluid cells were centrifuged and the cell pellets were resuspended in PBS, counted using a Coulter Counter (Coulter Electronics, Luton, UK), and visually differentiated after Giemsa staining. Bronchial lymph nodes (LN) were excised and cell suspensions were prepared (see below).

#### Cell culture

The culture medium used was RPMI-1640 supplemented with 10% FCS, 100 μg/mL streptomycin, and 100 IU/mL penicillin. Cell suspensions were made by pressing the LNs through a cell strainer (Falcon, Franklin Lakes, NJ, USA). Cells were counted using a Coulter Counter. LN cell suspensions were cultured at 10 ^6^ cells/mL culture medium with 5 μg/mL Concanavalin A (MP Biomedicals, Irvine, CA, USA) in 96-well tissue culture plates (Nunc, Roskilde, Denmark) for 24 h. Spleen cell suspensions were cultured at 10 ^6^ cells/mL culture medium with 1 mg/mL OVA in 96-well tissue culture plates (Nunc) for 120 h. Culture conditions were 37 °C in a humidified atmosphere containing 5% CO _2_ .

#### Serum Ig and cytokine measurements

OVA-specific IgE and OVA-specific IgG1 were measured using an ELISA (Cayman Chemicals, Sanbio, Uden, the Netherlands). A 10-plex panel containing beads for mouse IL-1β, IL-4, IL-5, IL-17A, IFN-γ, MCP-1, and TNF-α (Merck, Darmstadt, Germany) was used.

#### Statistics

Statistical analysis of animal weights, BALF cell percentages, spleen and LN weights and cellularity, serum IgE and IgG1 levels, and cytokine production was performed using the independent-samples t-test (SPSS Inc., Chicago, IL, USA). Number of animals per group = 6.

## Additional file

Additional file 1: Table S1. Effect of exposure to TiO_2_ nanoparticles on viability (WST-1). **Table S2.** Effect of exposure to TiO_2_ nanoparticles on viability (live-dead). **Table S3.** Effect of exposure to TiO_2_ nanoparticles on CD83 expression. **Table S4.** Effect of exposure to TiO_2_ nanoparticles on CD86 expression. **Table S5.** Effect of exposure to TiO_2_ nanoparticles on IL-12p40 production. **Table S6.** Effect of exposure to TiO_2_ nanoparticles on TNF-α production. **Table S7.** Effect of exposure to TiO_2_ nanoparticles on IL-6 production. (DOCX 36 kb)

## Abbreviations

NP: Nanoparticles
OVA: Ovalbumin
