# Supplementary material for: The crystal structure of titanium dioxide nanoparticles influences immune activity in vitro and in vivo
Source: Part Fibre Toxicol. 2018 Jan 30;15:9. doi: 10.1186/s12989-018-0245-5 (PMC5791356; doi:10.1186/s12989-018-0245-5)
Supplement: Additional file 1: Table S1. — Effect of exposure to TiO2 nanoparticles on viability (WST-1). Table S2. Effect of exposure to TiO2 nanoparticles on viability (live-dead). Table S3. Effect of exposure to TiO2 nanoparticles on CD83 expression. Table S4. Effect of exposure to TiO2 nanoparticles on CD86 expression. Table S5. Effect of exposure to TiO2 nanoparticles on IL-12p40 production. Table S6. Effect of exposure to TiO2 nanoparticles on TNF-α production. Table S7. Effect of exposure to TiO2 nanoparticles on IL-6 production. (DOCX 36 kb) [file 12989_2018_245_MOESM1_ESM.docx]

# Additional file 1

| crystal form | primary size (nm) | coating | manufacturer | WST-1 (%) |
| --- | --- | --- | --- | --- |
| anatase | 20 | none | Io-Li-Tec | 184 |
| rutile | 20-40 | SiO_2_ | Io-Li-Tec | 176 |
| anat./rut. | 10-30 | none | Skyspring | 149 |
| rutile | 20-40 | SiO_2_ | Skyspring | 136 |
| anatase | 10-25 | none | Skyspring | 131 |
| anatase | 10-25 | none | Io-Li-Tec | 131 |
| rutile | 10-30 | none | Skyspring | 130 |
| rutile | 20-40 | silicon oil | Io-Li-Tec | 127 |
| rutile | 10-30 | none | Io-Li-Tec | 124 |
| rutile | 200 | none | Io-Li-Tec | 119 |
| rutile | 20-40 | Al_2_O_3_ | Skyspring | 112 |
| rutile | 20 | hydrophobic | JRC | 107 |
| anatase | 20 | none | JRC | 103 |
| rutile | 20 | hydrophilic | JRC | 100 |

**Table S1** Effect of exposure to nanoparticles on viability (WST-1)

| crystal form | primary size (nm) | coating | manufacturer | Live-dead (%) |
| --- | --- | --- | --- | --- |
| rutile | 10-30 | none | Skyspring | 161 |
| anatase | 20 | none | JRC | 138 |
| rutile | 20-40 | SiO_2_ | Io-Li-Tec | 130 |
| anatase | 10-25 | none | Io-Li-Tec | 124 |
| rutile | 200 | none | Io-Li-Tec | 114 |
| rutile | 20 | hydrophobic | JRC | 107 |
| anatase | 10-25 | none | Skyspring | 105 |
| rutile | 10-30 | none | Io-Li-Tec | 92 |
| rutile | 20 | hydrophilic | JRC | 85 |
| anatase | 20 | none | Io-Li-Tec | 85 |
| anat./rut. | 10-30 | none | Skyspring | 77 |
| rutile | 20-40 | silicon oil | Io-Li-Tec | 74 |
| rutile | 20-40 | SiO_2_ | Skyspring | 59 |
| rutile | 20-40 | Al_2_O_3_ | Skyspring | 54 |

**Table S2** Effect of exposure to nanoparticles on viability (live-dead)

| crystal form | primary size (nm) | coating | manufacturer | CD83 induction/reduction |
| --- | --- | --- | --- | --- |
| anat./rut. | 10-30 | none | Skyspring | 3.12 |
| anatase | 10-25 | none | Skyspring | 2.28 |
| anatase | 20 | none | JRC | 2.06 |
| anatase | 10-25 | none | Io-Li-Tec | 1.83 |
| anatase | 20 | none | Io-Li-Tec | 1.83 |
| rutile | 20-40 | SiO_2_ | Skyspring | 1.47 |
| rutile | 10-30 | none | Skyspring | 1.15 |
| rutile | 20-40 | Al_2_O_3_ | Skyspring | 1.12 |
| rutile | 200 | none | Io-Li-Tec | 0.93 |
| rutile | 20-40 | SiO_2_ | Io-Li-Tec | 0.89 |
| rutile | 20 | hydrophilic | JRC | 0.84 |
| rutile | 20 | hydrophobic | JRC | 0.82 |
| rutile | 20-40 | silicon oil | Io-Li-Tec | 0.76 |
| rutile | 10-30 | none | Io-Li-Tec | 0.70 |

**Table S3** Effect of exposure to TiO_2_ nanoparticles on CD83 expression

| crystal form | primary size (nm) | coating | manufacturer | CD86 induction |
| --- | --- | --- | --- | --- |
| anat./rut. | 10-30 | no | Skyspring | 2.47 |
| anatase | 20 | no | JRC | 2.27 |
| rutile | 20-40 | SiO_2_ | Skyspring | 2.14 |
| anatase | 20 | no | Io-Li-Tec | 1.84 |
| anatase | 10-25 | no | Io-Li-Tec | 1.80 |
| anatase | 10-25 | no | Skyspring | 1.78 |
| rutile | 20-40 | Al_2_O_3_ | Skyspring | 1.36 |
| rutile | 10-30 | no | Skyspring | 1.33 |
| rutile | 200 | no | Io-Li-Tec | 1.30 |
| rutile | 10-30 | no | Io-Li-Tec | 1.25 |
| rutile | 20 | hydrophobic | JRC | 1.24 |
| rutile | 20 | hydrophilic | JRC | 1.18 |
| rutile | 20-40 | SiO_2_ | Io-Li-Tec | 1.12 |
| rutile | 20-40 | silicon oil | Io-Li-Tec | 1.04 |

**Table S4** Effect of exposure to TiO_2_ nanoparticles on CD86 expression

| crystal form | primary size (nm) | coating | manufacturer | IL-12p40  Induction/reduction |
| --- | --- | --- | --- | --- |
| anatase | 20 | none | JRC | 2.74 |
| anatase | 10-25 | none | Io-Li-Tec | 1.86 |
| anat./rut. | 10-30 | none | Skyspring | 1.69 |
| anatase | 20 | none | Io-Li-Tec | 1.63 |
| anatase | 10-25 | none | Skyspring | 1.45 |
| rutile | 20-40 | Al_2_O_3_ | Skyspring | 1.08 |
| rutile | 200 | none | Io-Li-Tec | 0.97 |
| rutile | 10-30 | none | Skyspring | 0.94 |
| rutile | 20-40 | SiO_2_ | Skyspring | 0.86 |
| rutile | 20 | hydrophobic | JRC | 0.41 |
| rutile | 10-30 | none | Io-Li-Tec | 0.40 |
| rutile | 20-40 | silicon oil | Io-Li-Tec | 0.32 |
| rutile | 20-40 | SiO_2_ | Io-Li-Tec | 0.30 |
| rutile | 20 | hydrophilic | JRC | 0.12 |

**Table S5** Effect of exposure to TiO_2_ nanoparticles on IL-12p40 production

| crystal form | primary size (nm) | coating | manufacturer | TNF-α induction/reduction |
| --- | --- | --- | --- | --- |
| anatase | 20 | no | Io-Li-Tec | 7.01 |
| anatase | 20 | no | JRC | 6.99 |
| rutile | 20-40 | SiO_2_ | Io-Li-Tec | 4.50 |
| rutile | 200 | no | Io-Li-Tec | 3.71 |
| anat./rut. | 10-30 | no | Skyspring | 3.32 |
| anatase | 10-25 | no | Skyspring | 2.33 |
| rutile | 20-40 | silicon oil | Io-Li-Tec | 2.27 |
| rutile | 10-30 | no | Io-Li-Tec | 2.05 |
| rutile | 20 | hydrophobic | JRC | 1.74 |
| anatase | 10-25 | no | Io-Li-Tec | 1.72 |
| rutile | 20-40 | Al_2_O_3_ | Skyspring | 1.40 |
| rutile | 10-30 | no | Skyspring | 1.27 |
| rutile | 20-40 | SiO_2_ | Skyspring | 1.13 |
| rutile | 20 | hydrophilic | JRC | 0.69 |

**Table S6** Effect of exposure to TiO_2_ nanoparticles on TNF-α production

| crystal form | primary size (nm) | coating | manufacturer | IL-6  Induction/reduction |
| --- | --- | --- | --- | --- |
| anatase | 20 | no | Io-Li-Tec | 1.61 |
| rutile | 200 | no | Io-Li-Tec | 1.43 |
| anatase | 20 | no | JRC | 1.11 |
| rutile | 10-30 | no | Skyspring | 0.93 |
| rutile | 20-40 | SiO_2_ | Io-Li-Tec | 0.90 |
| rutile | 20-40 | silicon oil | Io-Li-Tec | 0.90 |
| rutile | 10-30 | no | Io-Li-Tec | 0.82 |
| rutile | 20-40 | SiO_2_ | Skyspring | 0.78 |
| rutile | 20-40 | Al_2_O_3_ | Skyspring | 0.76 |
| anat./rut. | 10-30 | no | Skyspring | 0.72 |
| anatase | 10-25 | no | Skyspring | 0.56 |
| anatase | 10-25 | no | Io-Li-Tec | 0.52 |
| rutile | 20 | hydrophobic | JRC | 0.49 |
| rutile | 20 | hydrophilic | JRC | 0.31 |

**Table S7** Effect of exposure to TiO_2_ nanoparticles on IL-6 production
